# Thyrotoxicosis Associated with a Hypopharyngeal Toxic Nodular Thyroid

**DOI:** 10.1155/2017/5128563

**Published:** 2017-11-02

**Authors:** S. Ali Imran, Adam Hinchey, Rob Hart, Martin Bullock, Andrew Ross, Steven Burrell

**Affiliations:** ^1^Division of Endocrinology and Metabolism, Department of Medicine, Dalhousie University, Halifax, NS, Canada; ^2^Dalhousie Medical School, Halifax, NS, Canada; ^3^Division of Otolaryngology, Department of Surgery, Dalhousie University, Halifax, NS, Canada; ^4^Department of Pathology, Dalhousie University, Halifax, NS, Canada; ^5^Division of Nuclear Medicine, Department of Diagnostic Radiology, Dalhousie University, Halifax, NS, Canada

## Abstract

Ectopic thyroid is a rare developmental anomaly which may be either asymptomatic or present with thyroid dysfunction as well as pressure symptoms. Here we present a novel case of thyrotoxicosis associated with a hypopharyngeal multinodular thyroid in a female. Removal of the ectopic thyroid led to normalization of the thyroid status.

## 1. Case Description

A 32-year-old female was referred to the endocrine clinic for assessment of hyperthyroidism. She was previously known to have mild depression that was controlled on Cipralex 10 mg daily. She had originally gone to her family physician for frequent palpitations and excessive sweating for the past 3 months. Preliminary work-up showed thyroid stimulating hormone (TSH) of <0.01 mIU/L (normal: 0.35–5.50) and serum free thyroxine (fT4) of 20 pmol/L (9.5–19.0). The family physician prescribed methimazole, 5 mg twice a day, and 8 weeks later her TSH became 6.01 mIU/L and serum fT4 dropped to 9.0 pmol/L. Methimazole was discontinued and she remained biochemically euthyroid until a follow-up blood test around 9 months later showed TSH of <0.01 mIU/L, fT4 of 17.4, and free triiodothyronine (fT3) of 7.2 pmol/L (normal: 3.5–6.5) at which point she was referred to endocrinology.

**Figure 1:**
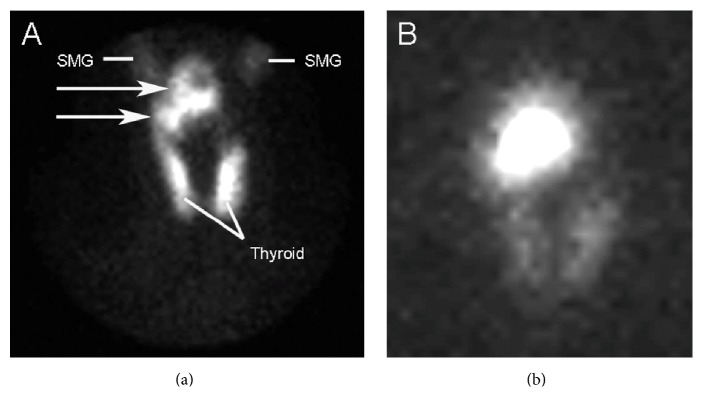
(a) Anterior view from initial ^99m^Tc-pertechnetate thyroid scan. There is heterogeneous uptake in a lobulated mass (arrows) superior to the right lobe of the thyroid. Uptake in the thyroid gland is relatively homogeneous. There is normal physiologic uptake in the submandibular glands (SMG). ^131^Iodine scan (b) demonstrates intense radioiodine uptake in the mass.

**Figure 2:**
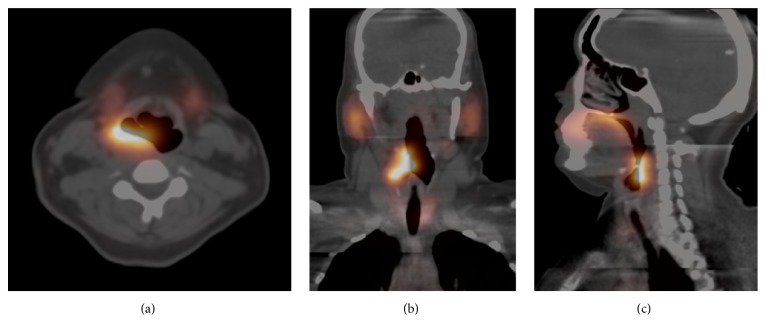
SPECT-CT images from the second ^99m^Tc-pertechnetate thyroid scan ((a) axial, (b) coronal, and (c) sagittal) localize the mass to the posterior right hypopharynx.

She denied any family history of thyroid disorders and, on initial assessment, her pulse was 82/minute (regular), blood pressure was 110/84, she had mild tremor and moist skin, and her reflexes were brisk (grade 3). There was no evidence of thyroid associated ophthalmopathy. Methimazole 5 mg twice a day was again initiated and the preliminary investigations revealed that thyroid receptor antibody was <1 IU/L (normal < 1) and antithyroid peroxidase antibody was < 10 IU/mL (normal ≤ 40). A ^99m^Tc-pertechnetate thyroid scan was performed (Figure 1(a)) after discontinuing methimazole for 4 days, which revealed heterogeneous uptake in a large lobulated mass superior to the right lobe of the thyroid. Uptake throughout the thyroid itself was relatively homogenous. The 6-hour radioiodine (^131^Iodine) uptake was measured at 27.1% (normal < 20%) but may have been underestimated as counts from the mass above the thyroid may not have been fully detected by the probe. Her thyroid indices normalized with methimazole 5 mg daily, which was continued for one year and then stopped. However, within few weeks of stopping methimazole she experienced recurrence of thyrotoxic symptoms including palpitations, excessive sweating, and anxiety and repeat testing showed suppression of serum TSH to <0.01 mIU/L with fT4 being 16.1 pmol/. ^99m^Tc-pertechnetate was repeated which was similar in appearance (not shown). This time SPECT-CT (Single Photon Emission Computed Tomography-Computed Tomography) imaging was also performed to better localize the mass, revealing it to be in the right posterior hypopharynx, measuring up to 4 cm cranial-caudal (Figure 2). A thyroid ultrasound was performed which showed a 9.0 × 9.5 CM slightly heterogeneous mass with internal vascularity just above the superior aspect of the right thyroid with no other focal lesion in the right lobe and a small 5 mm mixed echogenicity nodule in the lower left lobe. An ^131^Iodine scan was then performed (Figure 1(b)) that revealed intense radioiodine uptake in the mass, confirming that it was indeed thyroidal in origin. The uptake in the mass was more intense than the uptake in the thyroid gland, in keeping with a hyperfunctioning (“hot”) nodule.

**Figure 3:**
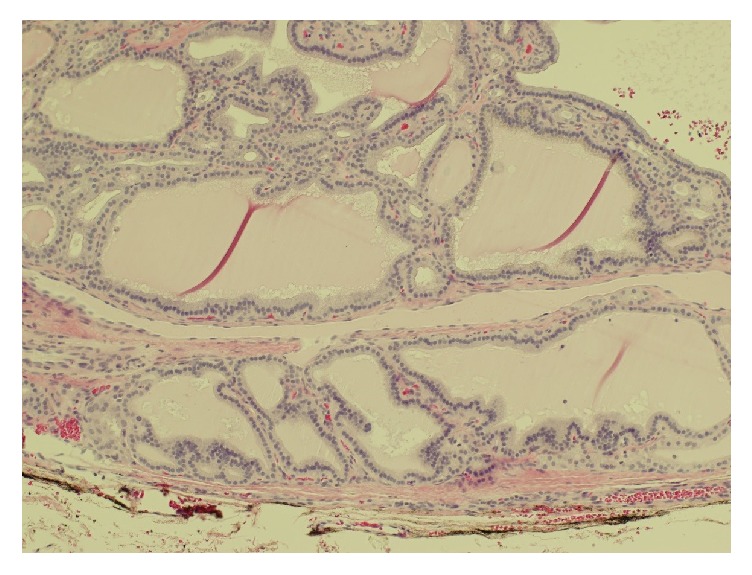
Photomicrograph of the edge of the dominant nodule, showing irregularly dilated follicles with pale colloid and subtle infoldings of the follicular epithelium. The follicular cells are uniform and evenly spaced, without features of papillary thyroid carcinoma (H&E, 100x).

The patient did not complain of any symptoms due to mass effect of the lesion. The finding and management options including continuing medical therapy with methimazole, radioiodine therapy, and surgical excision were discussed with the patient, who opted for surgical excision of the mass. She was maintained in a euthyroid state on methimazole and surgical excision of the lateral hypopharyngeal mass was conducted. Given the uncommon location, care was taken to avoid injury to the superior laryngeal nerve, which was proven normal on postoperative endoscopy. The surgical specimen consisted of a 16 g, 5.3 cm mass of thyroid tissue. Some normal thyroid parenchyma was present that contained several small hyperplastic nodules, but the specimen was dominated by an attached 3.5 cm circumscribed nodule of hyperplastic thyroid tissue. This consisted of a mixed population of microfollicles, macrofollicles, and small cysts containing pale colloid. Focal papillary infoldings of hyperplastic follicular cells extended into the dilated follicles (Figure 3). The nodule was partially fibrotic and focally calcified. There was no significant inflammation in the nodule or the adjacent normal thyroid parenchyma. There was no evidence of malignancy. Methimazole was discontinued after surgery and the patient remains biochemically euthyroid.

## 2. Discussion

Ectopic thyroid tissue is a rare developmental abnormality involving defects in the embryogenesis of the thyroid gland during its embryological descent. Its prevalence is reported to be approximately 1 per 100,000–300,000 people but it is likely much commoner as the autopsy studies have suggested the prevalence being as high as 7–10% [1]. In the reported larger series, ectopia of the thyroid is commoner in females [2, 3]. The thyroid gland is derived from the endoderm and an understanding of the development of the gland can aid in identifying typical locations where ectopic thyroid tissue can develop. The thyroid is one of the earliest glands to develop with the formation of the medial anlage on gestation days 16 and 17. The expanding thyroid remains attached to the pharyngeal floor by the stalk called the thyroglossal duct. The medial portion of the thyroid is associated with the developing heart and with the descent of the heart the thyroid is pulled caudally into its position at the base of the neck with the consequent elongation of the thyroglossal duct which generally degenerates. Although the ectopic thyroid tissue can develop anywhere along the developmental pathway, the base of the tongue remains the most common site accounting for up to 90% of the cases of ectopic thyroid [4]. However, ectopic thyroid tissue has been reported around the hyoid bone, infrahyoid and lateral portions of the neck, and rarely in peripheral sites such as the mediastinum, adrenal glands, and the duodenum.

Symptoms of the ectopic thyroid can vary depending upon its location. The commonest location is the base of the tongue where it can present with dysphonia, dysphagia, cough, sleep apnea, and rarely with obstruction [5, 6]. Other sites include submandibular thyroid which can present as a localized swelling in the carotid triangle, whereas intratracheal thyroid has also been described which can present with cough, dyspnea, hemoptysis, and stridor. Rarely reported intrathoracic and intracardiac thyroid can present with signs of obstruction, chest pain, and palpitations [7, 8]. Neoplasia of the ectopic thyroid tissue has also previously been described. Typically, neoplasms of the thyroglossal duct tend to be papillary carcinoma; however, follicular thyroid cancer has also been described [9]. Thyrotoxicosis associated with ectopic thyroid tissue has also been described as a result of retrotracheal and intrathoracic toxic nodules and lingual thyroid as well as submandibular thyroid [10–13]. To our knowledge no case of an ectopic pharyngeal multinodular thyroid has been described previously, particularly in the setting of an otherwise normally positioned thyroid gland.

Although the most appropriate therapeutic option for an ectopic thyroid remains unclear, most reported cases underwent surgery. Surgical excision would obviously be required in case of obstructive symptoms; yet in asymptomatic cases nonsurgical follow-up may be sufficient. Others have reported I-131 ablation therapy for ectopic thyroid [14]; however, it is suggested that a significantly higher dose of I-131 [15] may be needed for ablation in such cases. Therefore, ablation therapy should be carefully assessed in younger individuals.

In summary, this case highlights an unusual case of thyrotoxicosis due to an ectopic hyperfunctioning thyroid mass in the lateral hypopharynx, localized preoperatively with SPECT-CT imaging. The pathology showed a multinodular thyroid.
